# Association of MLL3 and TGF-β signaling gene polymorphisms with the susceptibility and prognostic outcomes of Stanford type B aortic dissection: MLL3 with TGF-β signal pathway association with Stanford type B AD

**DOI:** 10.1186/s12872-023-03287-8

**Published:** 2023-05-24

**Authors:** Qinghua Yuan, Yafei Chang, Peipei Jiang, Ling Sun, Yitong Ma, Xiang Ma

**Affiliations:** 1 Department of Cardiology, The Seventh Affiliated Hospital of Sun Yat-sen University, Shenzhen, China; 2 Faculty of Forensic Medicine, Zhongshan School of Medicine, Sun Yat-sen University, Guangzhou, China; 3 Department of Geriatrics, The Fourth People’s Hospital of Urumqi City, Urumqi, China; 4 Department of Cardiology, Fuyang Tumor hospital, Fuyang, China; 5 Department of Cardiology, First Affiliated Hospital of Xinjiang Medical University, Urumqi, China; 6 First Affiliated Hospital of Xinjiang Medical University, 137 Liyushan South Road, Urumqi, 830054 China

**Keywords:** Stanford type B AD, MLL3, TGFβ signal pathway

## Abstract

**Objective:**

This study aims to investigate the association of lysine methyltransferase 2 C (MLL3) and transforming growth factor β (TGF-β) signaling-related gene polymorphisms with the susceptibility of Stanford type B aortic dissection (AD) and its clinical prognostic outcomes. The methods involved investigating the MLL3 (rs10244604, rs6963460, rs1137721), TGFβ1 (rs1800469), TGFβ2 (rs900), TGFR1 (rs1626340) and TGFR2 (rs4522809) gene polymorphisms. Logistic regression was performed to investigate the association between 7 single nucleotide gene polymorphisms (SNPs) and Stanford type B aortic dissection. The GMDR software was used to analyze gene-gene and gene-environment interactions. The odds ratio (OR) with a 95% confidence interval (CI) was employed to evaluate the association of genes and Stanford type B AD risk.

**Results:**

Genotypes and allele distributions in the case and control groups showed significant differences (P < 0.05). Logistic regression has shown that the Stanford Type B AD risk was highest in individuals with the rs1137721 CT genotype (OR = 4.33, 95% CI = 1.51–12.40). Additionally, WBC, drinking, hypertension, triglycerides (TG), and low-density lipoprotein (LDL-C) were independent risk factors for Stanford Type B AD. Logistic regression showed that the Stanford Type B AD risk was highest in individuals with the MLL3 (rs1137721)-TT + CT and TGFβ1 (rs4522809)-AA genotype (OR = 6.72, 95% CI = 1.56–29.84), and lowest in those with the MLL3 (rs1137721)-CC and TGFβ1 (rs4522809)-AA + GG genotype (OR = 4.38, 95% CI = 0.92–20.83). However, the 55-month median long-term follow-up did not show statistical significance.

**Conclusion:**

Carriers of both TT + CT of MLL3 (rs1137721) and AA of TGFβ1 (rs4522809) polymorphisms may be closely related to the development of Stanford type B AD. MLL3 (rs1137721), WBC, and TG/TC were found to be associated with the morbidity of Stanford type B AD. MLL3 (KMT2C) is associated with the TGF-β signaling pathway protein. The risk of Stanford type B AD is related to the interactions of gene-gene and gene-environment.

## Introduction

The incident of Type B Aortic dissection (AD) has significant morbidity and mortality [1, 2]. AD develops due to multiple factors, including gene-related mutations and environmental factors [3, 4]. The complicated pathogenesis of AD is accompanied by the structural weakness of aortic connective tissue and an increased TGF-β signaling pathway [5, 6]. Inflammatory T lymphocytes and macrophages infiltrate the Aortic media and adventitia [7, 8]. Epigenetic modifications can also affect the development of AD [9, 10].

Transforming growth factor-β (TGF-β) signaling plays a critical role in the development and maintenance of vasculature [11, 12]. Mutations in the genes encoding TGF-β receptors, TGFBR1 and TGFBR2, are commonly encountered [13], as are their cognate ligands, such as TGF-β2[14]. Additionally, an intracellular effector of TGF-β signaling is also present [15].

Epigenetic modifications can significantly affect the development or progression of many cardiovascular diseases. Lysine methyltransferase 2 C (KMT2C, Mll3) is an H3K4 methyltransferase that participates in adipogenesis. In addition to its association with Stanford type B AD, the MLL3/4 complex has been found to interact with the TGF-β signal pathway in lipid metabolism-related GO terms [16]. These findings suggest that MLL3 may play an important role in the regulation of the TGF-β signal pathway in both normal and pathological conditions. In the regulation of transcription in lipid accumulation, MLL3 and MLL4 have distinct and critical roles and can increase energy expenditure and bile acid (BA) levels [17, 18]. However, the relationship between MLL3 polymorphisms and aortic dissection is not known. Understanding the biology of AD-related disorders remains insufficient. The study aimed to investigate the impact of MLL3 and TGF-β signal pathway, and synergistic interaction between MLL3 and TGF-β signal pathway, on Stanford type B AD risk.

## Methods

### Sample design

We recruited people who were in the hospital at the First Affiliated Hospital of Xinjiang Medical University from 2012 to 2016. All of them underwent Computed Tomography Angiography (CTA). We enrolled 382 participants (AD patients: 175; Control groups: 197). AD patients who underwent Thoracic Endovascular Aortic Repair (TEVAR) were recruited as the study group. The control patients who underwent Coronary Angiography or Computed Tomography Angiography (CTA) were confirmed to have no coronary disease. The exclusion criteria were as follows: Patients who had a bicuspid aortic valve; patients who had an aortic disease, such as Marfan syndrome and aortic coarctation; patients who had incomplete data; patients who were diagnosed with coronary artery disease or cardiomyopathy. Participants were of any ethnicity except for Han Chinese. The sex ratio of patients and the control group was 1:1. The age range was from 20 to 86 years, with a mean ± SD age of 53.78 ± 11.91 years (51.18 ± 12.01 years for AD patients and 55.87 ± 11.43 years for the control group). Demographic data included information about the presence of traditional heart disease risk factors, including hypertension, smoking, alcohol consumption, and diabetes mellitus. Blood samples were obtained to measure basal fasting serum concentrations of white blood cell, creatinine, total cholesterol, high-density lipoprotein cholesterol, low-density lipoprotein cholesterol, glucose, triglycerides, and fasting blood glucose (FBG). The measurements were conducted by the clinical laboratory department of the First Affiliated Hospital of Xinjiang Medical University with a biochemical analyzer (Dimension AR/AVL Clinical Chemistry System, Newark, NJ, USA). Left ventricular diastolic diameter (LVDd) and left ventricular systolic diameter (LVSd) were two-dimensionally measured using Hp5500 the ultrasonocardiograph. Left ventricular ejection fraction (EF) was calculated based on the formula of EF=(LVDd-LVSd)/ LVDd.

### Genotyping

The SNPs for the human MLL3 gene listed in the National Center for Biotechnology Information SNP database (http://www.ncbi.nlm.nih.gov/SNP). We screened data for the Tag SNPs in the International HapMap Project website (http://www.hapmap.org/). We used Haploview 4.2 software (Harvard University, Cambridge, MA, USA) and the HapMap phase II database obtained three tagging SNPs [rs10244604 (g.152,328,895 A > G), rs6963460 (g.152,187,985 A > G), rs1137721 (g.152,301,320 C > G, T)] for the Chinese Han subjects using a minor allele frequency (MAF) ≥ 0.05 and linkage disequilibrium patterns with r2 ≥ 0.8 as a cutoff. Genomic DNA was extracted from peripheral blood leukocytes using a DNA extraction kit (Beijing Biotech Co. Ltd. Beijing, China). Genotyping was performed using the TaqMan® SNP Genotyping Assay (Applied Biosystems Inc., Foster City, CA, USA) as described previously [19]. Two researchers without the knowledge of case or control status blindly conducted all assays. Additionally, approximately 10% of the samples were randomly selected and retested, and the results were 100% concordant.

### Statistical analysis

Quantitative variables with normal distribution were presented as mean ± SD and analyzed using Student’s t-test or ANOVA test, while non-normal distribution was presented as median (interquartile range) and analyzed using Mann-Whitney U-test. Statistical significance was established at P < 0.05. Statistical analyses performed using SPSS software for Windows, version 20.0 (SPSS, Chicago, Illinois, USA). The gene interaction model was determined using MDR1.10 according to the standards of the largest cross-validation consistency coefficient and highest checking sample accuracy. Missing values were filled in by means/medians/modes.

### Survival analysis

Patients were followed up for a median of 55.7 (47.6–57.9) months. Kaplan-Meier survival analysis was performed to calculate the association between prognosis. The information was acquired from the records of their inpatients or outpatients or by telephone calls. The endpoint event was all-cause mortality.

### PPI Network

**Fig. 1:**
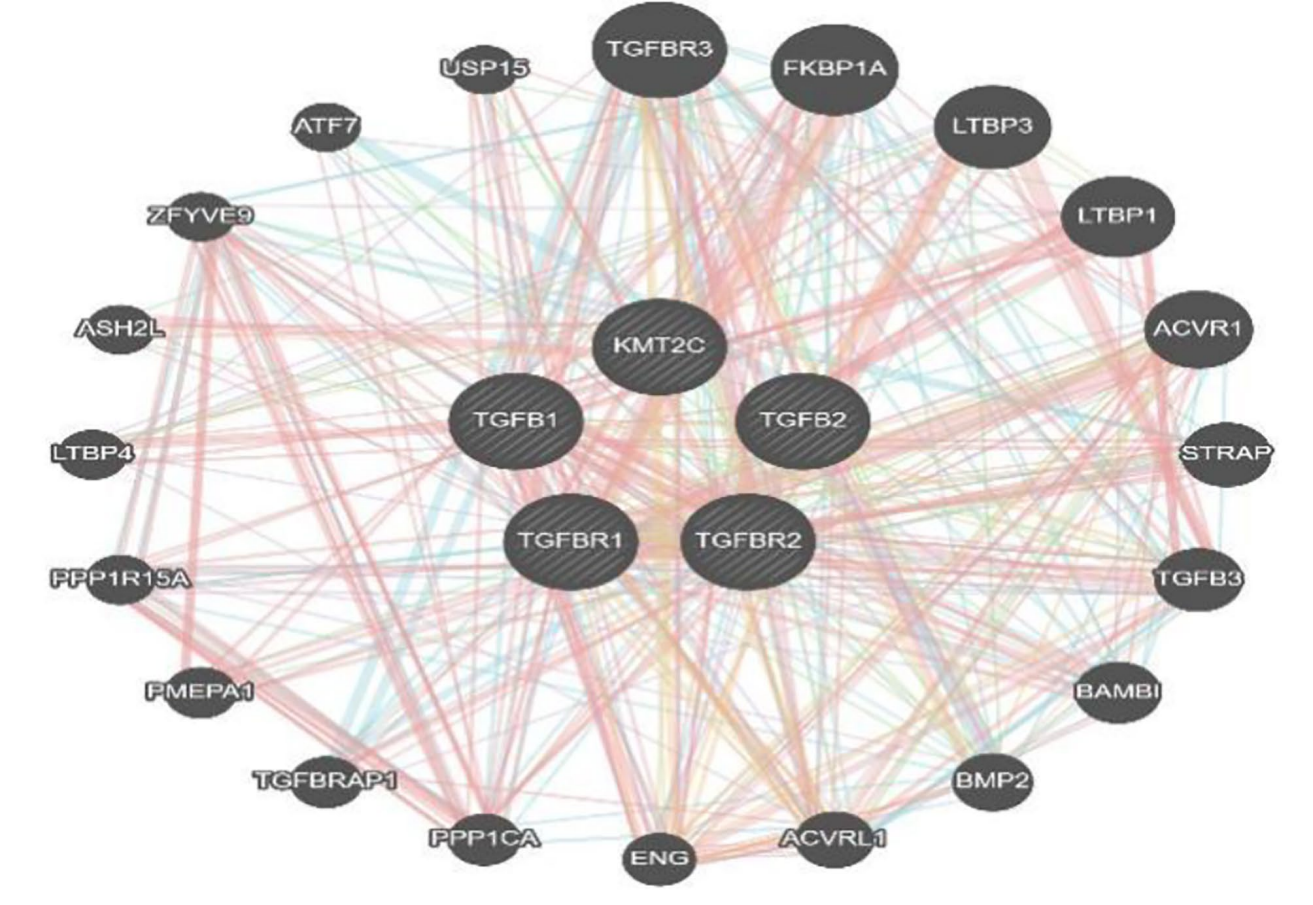
Protein-protein interaction (PPI), lysine methyltransferase 2 C (KMT2C, MLL3) associated with transforming growth factor beta (TGF-β) signal pathway protein

A Protein-protein interaction (PPI) network with 24 genes centered on MLL3(KMT2C) was constructed using Gene MANIA (Figure 1).

### Followed up

We conducted a 4-year clinical followed-up for case group. All follow-up results were acquired by telephone calls, outpatient records or readmission. The baseline demographic data, clinical and clinical endpoint events of the selected patients were recorded. The primary endpoint was death due to the recurrence of AD, and the secondary endpoint was hospitalization for chest pain recurrence.

## Results

### Comparison of clinical data

The differences in clinical data [hypertension, diabetes, smoking, drinking, age, and gender, Total cholesterol(TC), Triglycerides(TG), high-density lipoprotein(HDL), white blood cell(WBC), and Ejection fraction(EF)] between Stanford type B AD (158) and the control group (197) were analyzed. As shown in (Table 1), significant differences were found (P < 0.05), which suggested that clinical factors of smoking, drinking, hypertension, diabetes, age, Body mass index(BMI), TC, TG, Systolic blood pressure(SBP), Diastolic blood pressure(DBP), creatinine(Scr), WBC, Fasting blood glucose (FGB) were associated with Stanford type B AD susceptibility.

**Table 1:** Basic Characteristic of Participants Stratified According to with or without Stanford type B aortic dissection

Variables	Controls (n = 197)		AD cases (n = 158)	P
Gender (male)		157(44.2)	133(37.5)	0.33
Age (year)		55.87 ± 11.43	51.18 ± 12.01	< 0.01
BMI (kg/m^2^)		25.84 ± 3.08	27.10 ± 7.26	0.04
Smoking (%)		63(17.8)	94(26.6)	< 0.01
Drinking (%)		45(12.7)	90(25.5)	< 0.01
Hypertension (%)		83(23.4)	123(34.7)	< 0.01
Diabetes (%)		21(6)	10(2.9)	0.14
SBP(mmHg)		126.69 ± 16.63	151.30 ± 29.75	< 0.01
DBP(mmHg)		80.17 ± 32.11	87.05 ± 18.80	0.01
Scr (ummo/l)		74.34 ± 16.79	94.16 ± 100.76	< 0.01
TC(mmol/l)		4.19 ± 1.12	4.43 ± 2.37	0.20
TG(mmol/l)		1.73 ± 0.99	2.60 ± 4.82	0.01
HDL-C(mmol/l)		1.10 ± 0.32	2.05 ± 5.01	< 0.01
LDL-C(mmol/l)		2.67 ± 1.18	5.63 ± 13.56	< 0.01
WBC(10^9^/l)		6.68 ± 1.98	11.52 ± 4.10	< 0.01
FGB(mmol/l)		5.46 ± 1.85	10.79 ± 31.31	0.02

Body mass index(BMI), fasting blood glucose (FBG) white blood cell(WBC), creatinine(Scr), total cholesterol(TC), high-density lipoprotein cholesterol(HDL-C), low-density lipoprotein cholesterol(LDL), triglycerides(TG),Systolic blood pressure(SBP), Diastolic blood pressure(DBP);

### Genotype multiple logistic regression analyses

(Table 2) shows the frequencies of (MLL3, TGFB1, TGFB2, TGFBR1, TGFBR2) alleles and genotype within seven SNPs in cases and controls. We found that the variants in rs1137721, and rs1800469 were related to increased AD risk after covariant adjustment. AD risks were higher in carriers of the homozygous mutant of rs1137721 CT/CC OR (95%CI) was 4.33(1.51–12.40), and rs1800469 AA + AG/GG OR (95%CI) was 3.43(1.04–11.30).

**Table 2:** a Genotype and allele distributions in patients with Stanford type B aortic dissection and control subjects

SNPs	Genotypes and Alleles	Frequencies N (%)			OR(95%CI)	P
		Cases(158)		Controls(197)		
MLL3 rs10244604	AA	117(34.5)	159(46.9)		1.0	
	AG	26(7.7)	33(9.7)		1.13(0.44–2.94)	0.80
	GG	2(0.6)	2(0.6)		0.64(0.04–10.6)	0.76
	AA + AG/GG	143(42.2)	192(56.6)		1.59(0.1–26.1)	0.75
	A	260(38.3)	351(51.8)			
	G	30(4.4)	37(5.5)			
rs6963460	AA	92(28.0)	128(39.0)		1.0	
	AG	40(12.2)	52(15.9)		1.11(0.4–2.7)	0.83
	GG	5(1.5)	11(3.4)		1.07(0.17–6.73)	0.94
	AA + AG/GG	132(40.2)	180(54.9)		0.96(0.16–5.89)	0.97
	A	224(34.1)	308(47.0)			
	G	50(7.6)	74(11.3)			
rs1137721	CC	46(13.8)	75(22.5)		1.0	
	CT	81(24.3)	90(26.9)		4.33(1.51–12.40)	< 0.01
	TT	15(4.5)	27(8.1)		2.19(0.45–10.65)	0.33
	CC + CT/TT	127(38.0)	165(49.4)		1.18(0.30–4.63)	0.81
	C	173(25.9)	240(35.9)			
	T	111(16.6)	144(21.6)			

**Table 2:** b Genotype and allele distributions in patients with Stanford type B aortic dissection and control subjects

SNPs	Genotypes and Alleles	Frequencies N (%)		OR(95%CI)	P
		cases	controls		
TGFBR1 rs1626340	AA	22(6.9)	36(11.3)	1.0	
	AG	70(22.0)	67(21.1)	2.91(0.97–8.73)	0.06
	GG	53(16.7)	70(22.0)	0.67(0.20–2.25)	0.52
	AA + AG/GG	123(38.7)	137(43.1)	1.59(0.59–4.29)	0.36
	A	114(17.9)	139(21.9)		
	G	176(27.7)	207(32.5)		
TGFBR2 rs4522809	AA	67(19.7)	102(30.0)	1.0	
	AG	71(20.9)	80(23.5)	1.34(0.56–3.22)	0.51
	GG	8(2.4)	12(3.5)	0.66(0.11–3.85)	0.65
	AA + AG/GG	138(40.6)	182(53.5)	1.75(0.32–9.58)	0.52
	A	205(30.1)	284(41.8)		
	G	87(12.8)	104(15.3)		
TGFB1 rs1800469	AA	35(10.9)	49(15.2)	1.0	
	AG	65(20.2)	95(29.5)	1.15(0.41–3.22)	0.78
	GG	29(9.0)	49(15.2)	0.33(0.08–1.34)	0.12
	AA + AG/GG	100(31.1)	144(44.7)	3.43(1.04–11.30)	0.04
	A	135(21.0)	193(30.0)		
	G	123(19.1)	193(30.0)		
TGFB2 rs900	AA	10(2.9)	9(2.7)	1.0	
	AT	64(18.9)	81(23.9)	0.67(0.13–3.45)	0.62
	TT	71(20.9)	104(30.7)	0.59(0.11–3.14)	0.54
	AA + AT/TT	74(21.8)	90(26.5)	1.16(0.52–2.60)	0.70
	A	84(12.4)	99(14.6)		
	T	206(30.4)	289(42.6)		

Adjustment for age, alcohol consumption, smoking, SBP, DBP, Scr, FBG, TG, HDL-C, LDL-C, DM, HP, BMI and WBC.

Body mass index(BMI), fasting blood glucose (FBG) white blood cell(WBC), creatinine(Scr), total cholesterol(TC), high-density lipoprotein cholesterol(HDL-C), low-density lipoprotein cholesterol(LDL), triglycerides(TG),Systolic blood pressure(SBP), Diastolic blood pressure(DBP);

### GMDR analyses

GMDR model was used to screen the potential best interaction combination among SNPs within MLL3 and TGF-β. In (Table 3), we found that there was a significant gene-gene interaction between rs1137721 and rs4522809. In this model, the cross-validation consistency is 10/10 and the testing accuracy is 57.19%. Logistic regression indicated that participants with rs1137721-TT + CT and rs4522809-AA genotype (OR = 6.72, 95% CI = 1.56–29.84) have the highest Stanford type B AD risk, compared to participants with rs1137721-CC and rs4522809-AA + GG genotype (OR = 4.38, 95%CI = 0.92–20.83), after covariant adjustment (Table 4). Meanwhile, we also screen the potential best interaction combination among SNPs with the environment (Table 5), we found that there was a significant gene-environment interaction among rs1137721, TG, and TC, In this model, the cross-validation consistency is 10/10 and the training accuracy is 100%.

**Table 3:** Best gene–gene interaction models, as identified by GMDR

Locus no	Best combination	Cross-validation consistency	Testing accuracy	p- values*	CV consistency
2	rs1137721	0.5531	0.4532	0.9893	4/10
3	rs1137721 rs4522809	0.5864	0.5759	0.0010	10/10
4	rs6963460rs1822825rs1800469	0.6305	0.4855	0.9453	6/10

**Table 4:** Interaction between rs1137721 and rs4522809 on Stanford type B aortic dissection risk

rs1137721	rs4522809	OR (95% CI)	p- values a
CC	AA	1.0	
TT + CT	AG + GG	6.27(1.42–27.75)	0.016
CC	AG + GG	4.38(0.92–20.83)	0.064
TT + CT	AA	6.72(1.56–28.94)	0.010

**Table 5:** Best gene–environment interaction models, as identified by GMDR

Best combination	Training Bal.Acc	Testing Bal.Acc	p- values*	CV consistency
TC	0.9051	0.6768	0.0010	10/10
TG LDL	0.9977	NaN	0.0010	6/10
rs1137721 TG TC	1.0000	NaN	0.0010	10/10

total cholesterol(TC), low-density lipoprotein cholesterol(LDL), triglycerides(TG);

### PPI Network

Protein-protein interaction show MLL3(KMT2C) associated with TGF-β signal pathway protein. Correlation analysis was performed between aortic dissection and control group, showing that the five proteins closely related.

### Kaplan–Meier analysis

At the end of the study, 30 patients died due to the recurrence of Stanford type B.

**Fig. 2:**
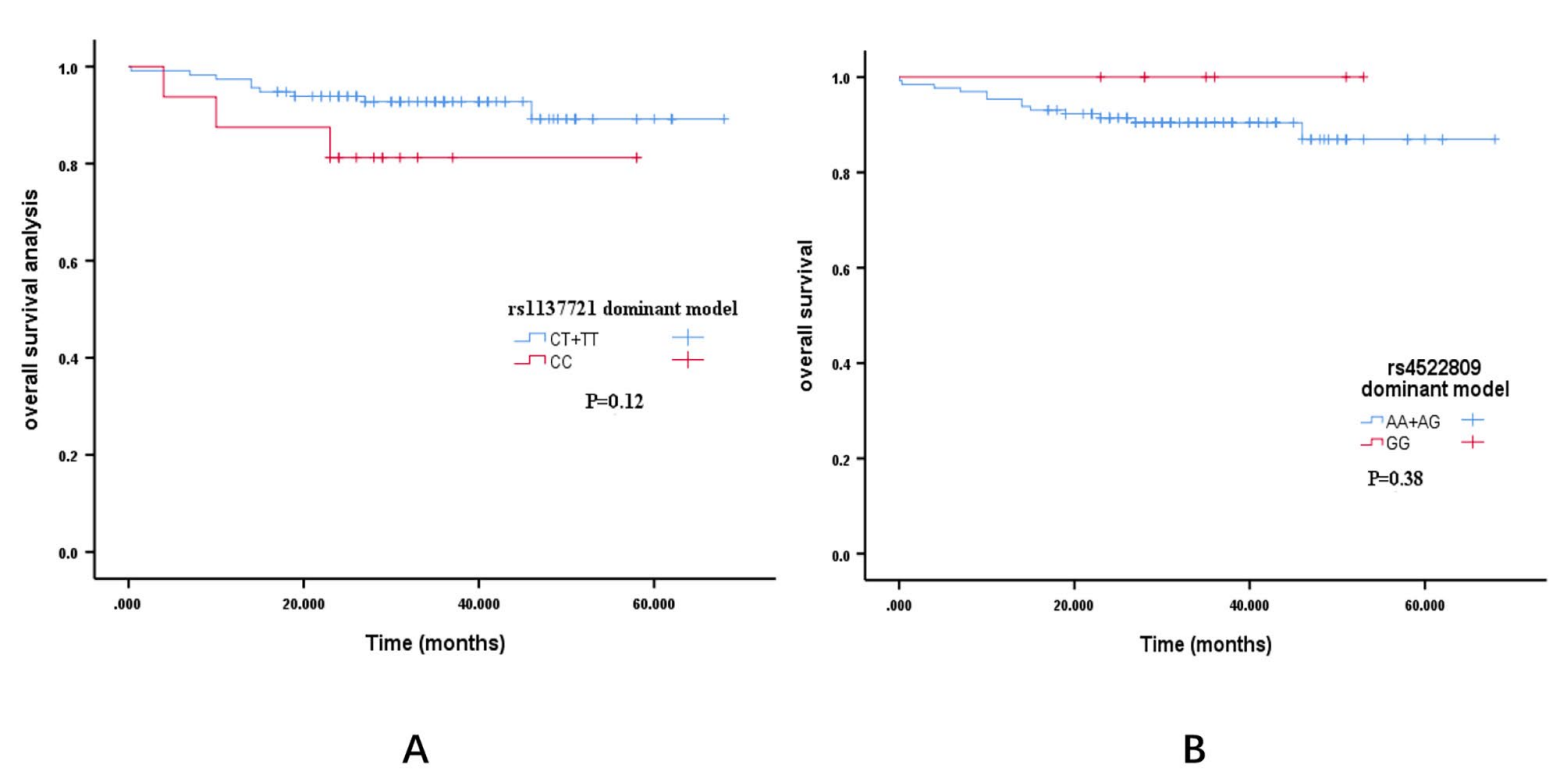
**A**: Kaplan–Meier analysis of the overall survival based on lysine methyltransferase 2 C (KMT2C, MLL3) rs1137721 dominant model; **B**: Kaplan–Meier analysis of the overall survival based on transforming growth factor receptor-2(TGFR2)rs4522809 dominant model;

AD. We use Kaplan–Meier method to analyze the association of tag SNPs and clinical outcomes in patients. The association of tag SNPs in patients with Stanford type B AD were assessed by the Kaplan-Meier test. Patients with these genotypes were associated with an increased mortality risk: dominant models of rs1137721 CT/CC, and rs4522809-AA genotype. No statistically significant differences were found between the association of mortality risk and genetic models of rs1137721 CT/CC, and rs4522809-AA genotype (Fig. 2).

## Discussion

In the current study based on the Chinese Han population, we found that variants in MLL3 (rs1137721) and TGFBR2 (rs4522809) were associated with a higher risk of Stanford type B AD. Inflammation reaction and lipid metabolism were also associated with Stanford type B AD. Traditional risk factors such as smoking, drinking, hypertension, diabetes, SBP, and DBP were identified. Meanwhile, HDL-C, LDL-C, and WBC were independent risk factors. Moreover, there exist MLL3-TGF-β pathway interactions among these risk factors for Stanford type B AD.

Based on our study, it indicates that serum lipid composition plays a critical role in aortic aneurysm formation and AD development [17]. Lipid metabolite LDL-C was an independent risk factor for Stanford type B AD patients [20]. Similar to our study findings, TG, HDL-C, and LDL-C are associated with AD. Multiple logistic regression analysis showed that LDL-C had no statistical significance, but the risk ratio was [OR = 1.30 (0.90–1.88), P = 0.16]. The Histone 3 lysine 4 (H3K4) methyltransferase MLL3 regulates lipid metabolic processes, including decreased white fat mass, lipid accumulation in the brown adipose tissue and liver, improved glucose tolerance, increased energy expenditure, and bile acid (BA) levels [16–18, 21]. MLL3/MLL4 acts as a master enhancer epigenomic writer activated during adipogenesis [22]. The MLL3(rs1137721 CT) genotype has the highest risk ratio (OR = 4.33, 95%CI = 1.51–12.40). GMDR showed the best gene-gene interaction models (rs1137721 rs4522809) in affecting Stanford type B AD risk. GMDR also showed that environmental factors TG and TC, along with MLL3(rs1137721), worked together in affecting Stanford type B AD risk. These findings clarify that the MLL3 gene influences the occurrence of lipid metabolic effects in the development of Stanford type B AD. However, long-term follow-up showed no statistical significance for all-cause mortality.

As identified by increased TGF-β signaling, it contributes to the complicated pathogenesis of aortic aneurysm [15]. TGFβ1 rs1800469 can affect the TGF-β1 plasma levels located in the promoter region. Rs1800469 is also associated with heart diseases [23]. However, the genetic associations between rs1800469 and Stanford type B aortic dissection are still confounding. Two studies suggested that the rs1800469 base mutation was associated with the presence of Abdominal Aortic Aneurysms in a UK cohort and Chinese cohort [24, 25]. However, after adjusting for con-founders, this association was lost. Our study’s logistic regression analysis showed that the rs1800469 dominant model [OR = 3.43(1.04–11.30), P = 0.04] was an independent risk factor for Stanford type B AD patients.

The GMDR analysis confirmed that TGFBR2 rs4522809, smoking, dyslipidemia, and MLL3 rs1137721 likely work together to affect the risk of Stanford type B AD. Mutations in genes associated with the transforming growth factor-β (TGF-β) signaling pathway can cause syndromic Thoracic Aortic Dissections (TAAD), such as Marfan syndrome (MFS), Loeys-Dietz syndrome (LDS), and Shprintzen-Goldberg Syndrome (SGS), which can potentially affect the cardiovascular system [3, 4]. The cytokine transforming growth factor-b type II receptor (TGFBR2) is regulated by Fibrillin-1[26]. In Marfan patients, TGF-β levels are elevated, resulting from increased MMP activity and extracellular matrix breakdown [27]. Research on experimental aneurysms has repeatedly revealed the activity of the TGF-β pathway in Thoracic Aortic Aneurysms [28]. The additional evidence of human mutations in genes encoding effectors of canonical TGF-β signaling has led to the hypothesis that aberrant TGF-β signaling drives aneurysm progression [29].

Aortic dissection (AD) has been recognized to be associated with an inflammatory process [30]. Chronic inflammation of the adventitia, media, and intima was found to be increased in AD [31]. Our study also shows that WBC is associated with AD. Logistic regression analysis shows significant results. The GMDR analysis demonstrated that MLL3 rs1137721, drinking, WBC, hypertension, and diabetes mellitus probably work together to affect Stanford type B AD risk. These findings suggest that WBC, dyslipidemia, and the TGF-β pathway might influence Stanford type B AD formation via the vascular fibrotic process, but the specific mechanism is unknown.

There were several limitations worth considering in this study. First, the sample size was small. It was found that the available sample sizes of 158 Stanford type B AD cases and 197 controls had > 60% and 85% statistical power to detect ORs of ~ 1.5 and ~ 1.8 for the association of the risk alleles with Stanford type B AD. Therefore, the results require a sizable sample to verify. Second, the number of signaling pathway SNPs genotyped was limited. Seven polymorphisms were chosen, and one gene-environment interaction affecting Stanford type B AD risk was discovered. Several factors that influenced the presence of Stanford type B AD, such as WBC, type 2 diabetes, hypertension, and atherosclerotic diseases, were found to have potential interactions with Stanford type B AD risk. Therefore, a more comprehensive study of the complex correlation in Stanford type B AD needs to be conducted.

## Conclusion

This study is the first to report that TGFBR2 rs4522809 and MLL3 rs1137721 genetic polymorphisms might be associated with Stanford type B AD risk in the Chinese Han population. Furthermore, complex interactions between environmental factors and polymorphisms might contribute to the risk of Stanford type B AD. Due to the small sample size, the results should be considered preliminary and requiring extensive validation and replication in larger populations.

## Data Availability

The data that support the findings of this study are available on request from the corresponding author. The data are not publicly available due to privacy or ethical restrictions.
